# Commencement Exercises of the Buffalo Medical College

**Published:** 1867-02

**Authors:** 


### Editorial Department.

### Commencement Exercises of the Buffalo Medical College.

NAMES OF THE GRADUATING CLASS, ETC., ETC.

The Commencement exercises of the Medical Department of the University of Buffalo were held in St. James Hall, February 26th, and were attended by a large and highly appreciative audience. The charge to the graduates by Prof. James P. White, was marked by the practical discernment and sagacious insight of the distinguished speaker. His address was full of suggestion and advice, making it not only highly interesting to the large, popular audience, but valuable and instructive to the young men just assuming the duties of professional life.

Dr. White also gave an interesting historical account of the Buffalo Medical College, and of what it has accomplished, showing the steps by which it has gained its present permanent and elevated position, among similar institutions. We hope to be able to publish the Address entire in our next number, which will make further comment unnecessary.

Milton G. Potter, A. B., M. D., gave the Valedictory Address, which, for appropriateness and elegance of composition, could not be surpassed. It was delivered with ease and grace—was what may truthfully be called "eloquent and impressive."

The examinations for the Degree of Doctor in Medicine were made publicly before the Curators and Faculty, at the college edifice during the day, and were sustained by the class with unusual ability. In point of thorough qualification this class have never been equaled in this institution, and there was an evident pride in the Faculty, in being able to present before the Curators so large a number of well qualified young men, for their approval as graduates.

The following gentlemen received the Degree of Doctor in Medicine:

Edwin T. M. Hurlbut, Ridgeway, N. Y.
George Monroe Proctor. Shaversville, Ohio.
Edward C. W. O'Brien, Buffalo, N. Y.
John Osborne Palmer, Victor, N. Y.
James Knowlton Bentley, Butternuts, N. Y.
Albert Carrier Bunn, Morris, N. Y.
Hiram Parsons Carey, Kiantone, N. Y.
Edwin Harrison Farrington, Holland, N. Y.
Duncan Alexander Campbell, St. Catharines, C. W.
John Nichols, Buffalo, N. Y.
Lucius Baker Parmele, A. B., Alden, N. Y.
John Botsford Sabin, Allen Centre, N. Y.
Charles F. A. Nichell, Buffalo, N. Y.
Daniel Lester Shull, Dansville, N. Y.
John Snowdon Weber, Brandt, N. Y.
Frederick Charles Stevens, Fredonia, N. Y.
Samuel Smith Nesbitt, Rayson, Ill.
Charles Brother Schuyler, A. M., Buffalo, N. Y.
Walter Alexander Rose, St. Thomas, C. W.
John Henry Wheeldon, Cowlesville, N. Y.
William Nelson Martin, Cowlesville, N. Y.
Israel Pattison, Welland, C. W,
Collins Chidcy Cook, Bellevue, Ohio.
Ganson Webster Croff, Bethany Centre, N. Y.
Erasmus Darwin Hills, Schyler's Lake, N. Y.
Hiram Peak Merville, East Java, N. Y.
Francis Burwell McCormick, Pelee Island, C. W.
Henry Olin Mackres, Clymer, N. Y.
Ezra Pennell, Darien, N. Y.

Samuel Robert Q. Cochrane, Waterport, N. Y.
Mahlon Bainbridge Folwell, A. M., Buffalo, N. Y.
Milton Grosvenor Potter, A. B., Attica, N. Y.
Henry Reid Hopkins, Buffalo, N. Y.
Charles Stewart Sheldon, A. M., Madison, Wis.
Alonzo Pettit A. M., Buffalo, N. Y.
James Addison Lounsbury, Bethany Mills, N. Y.
Arthus Benson Aikin, Moravia, N. Y.
Henry Harrison Fish, Mecklenburg, N. Y.
Orlando Beebee Sherwood, Cayutaville, N. Y.
Byron H. Daggett, Girard, Pa.

The following gentlemen were appointed Curators of the Medical Department, viz:—Dr. J. W. Craig, Churchville, N. Y.; Dr. Wm. H. Fish, Mecklenburg, Schuyler Co., N. Y.; Dr. C. W. Saunders, Belfast, Alleghany Co., N. Y.

